# Can shelter dog observers score behavioural expressions consistently over time?

**DOI:** 10.1186/s13028-022-00654-x

**Published:** 2022-12-02

**Authors:** Solveig Marie Stubsjøen, Randi Oppermann Moe, Cicilie Johannessen, Maiken Larsen, Henriette Madsen, Karianne Muri

**Affiliations:** 1 Department of Animal Health, Welfare and Food Safety, Section for Terrestrial Animal Health and Welfare, Norwegian Veterinary Institute, Elizabeth Stephansens Vei 1, 1433 Ås, Norway; 2 Department of Production Animal Clinical Sciences, Faculty of Veterinary Medicine, Norwegian University of Life Sciences, Elizabeth Stephansens Vei 15, 1433 Ås, Norway

**Keywords:** Animal welfare, Behaviour, Fixed list of descriptors, Observer reliability, Qualitative behaviour assessment, Shelter dogs

## Abstract

A substantial number of dogs live in animal shelters worldwide. Stressors within the shelter environment can compromise their welfare, and scientific evaluations of feasible welfare assessment methods are therefore needed. Qualitative Behaviour Assessment (QBA) is a “whole-animal” approach used to assess welfare by observing animals’ expressive behaviour. To investigate whether observers can score dogs’ behavioural expressions consistently over time, this study replicated and extended previous research, by evaluating intra- and inter-observer reliability of QBA based on video recordings of shelter dogs. In Part I, nine veterinary nurse students received theoretical and practical training, and then scored 12 2 min video recordings of shelter dogs using a fixed list of behavioural descriptors. Three of the students undertook further practice and calibration using direct observations of dog behaviour in a local shelter. In Part II, the videos from Part I were scored by these three observers a second time, 15 months later. QBA data were analysed using principal component analysis (PCA), and reliability was assessed using Kendall’s coefficient of concordance (*W*). In Part I, the inter-observer reliability was high for both components (0.78 for PC1 and 0.85 for PC2). In Part II, the inter-observer reliability was very high and moderate for PC1 and PC2, respectively (0.90 for PC1 and 0.65 for PC2). The intra-observer reliability was high for both components (*W* ≥ 0.86). Our results indicate that the fixed list of behavioural descriptors for shelter dogs can be used reliably when assessing videos, and that observers can score dogs’ behavioural expressions consistently after a break of 15 months following the initial assessment. Nevertheless, the reduction in inter-observer-reliability of PC2 in Part II can indicate that some retraining and calibration may be required to avoid observer drift.

## Findings

Animal shelters exist worldwide, and a substantial number of dogs live in shelters for prolonged periods. The aim of shelters is to rehome animals and thereby improving their long-term welfare [1]. To ensure good welfare during their stay in the shelter, dogs should experience more positive (e.g., pleasure) than negative (e.g., fear, frustration) emotions [2]. There are many potential stressors within the shelter environment that can compromise the dogs’ welfare, such as unfamiliar sounds, smells, routines, and people [3]. Scientific evaluations of feasible welfare assessment methods for shelter dogs are therefore needed. Shelter staff are often under-resourced, and a quick, reliable method to monitor welfare in a potentially large number of dogs over time would therefore be of value.

Qualitative Behavioural Assessment (QBA) is a “whole-animal” approach, where observers assess the animals’ expressive behaviour by integrating and summarising the details of behaviour, posture, and movement (body language) in light of the context [1, 2, 4, 5]. The validity of QBA has been supported by studies in various livestock species, demonstrating significant associations with other behavioural and physiological measurements [5]. However, further research is needed to investigate the use of QBA to assess dog welfare. A high level of reliability is an essential requirement for any method used to assess animal welfare [6], and reliability is considered a prerequisite for validity [7]. Observer reliability can be measured within a single observer (intra-observer) and between multiple observers (inter-observer) [8].

In a previous study, Stubsjøen et al. [9] found a high inter-observer reliability when observers used a fixed list of descriptors to assess videos of shelter dogs. To evaluate the robustness of this finding, we replicated the study by using the same fixed list of descriptors and video recordings to reassess inter-observer reliability. In addition, we extended the previous study by assessing the long-term inter- and intra-observer reliability. Our aim was to investigate whether observers can score dogs’ behavioural expressions consistently over time.

A group of nine final (3rd) year veterinary nurse students at the Norwegian University of Life Sciences, Faculty of Veterinary Medicine, consented to participate in Part I of the study. The students had not received any training in the use of QBA prior to the introductory presentation given at the first video scoring session. However, three of these students did their final year research project as a part of this project and had been asked to read six scientific papers on QBA of dogs, sheep, and broiler chickens prior to this session [1, 2, 10–13].

On the test day, the nine students were trained using the same procedure as in the previous study [9]. The video recordings of dogs were obtained from a shelter in southern Hungary, in which about 250 dogs were kept [9]. The animals were stray dogs or brought in from private homes for animal welfare reasons. After the introduction (~ 1 h), the students were first shown three test videos and thereafter encouraged to discuss their interpretation of the dogs’ behavioural expressions and to compare their scoring results. Subsequently, the fixed list of 20 qualitative descriptors, which included definitions of each, was used by the observers (Part Ia: n = 9 observers, Part Ib: n = 3 observers) to score the 12 videos [9] (Table 1).

**Table 1:** Kendall’s coefficient of concordance (*W*) for principal components and individual behavioural terms used by observers in Part I (Part Ia: n = 9 observers, Part Ib: n = 3 observers), and Part II (n = 3 observers) to assess shelter dogs in 12 video clips

Variable	Part Ia	Part Ib	Part II
	*W* for all observers	*W* for 3 observers	*W* for 3 observers
PC1	0.78	0.79	0.90
PC2	0.85	0.91	0.65
Content	0.57	0.52	0.37
Uncomfortable	0.58	0.86	0.63
Playful	0.68	0.62	0.64
Depressed	0.67	0.71	0.59
Relaxed	0.59	0.53	0.49
Restless	0.63	0.82	0.51
Alert	0.65	0.70	0.54
Bored	0.53	0.76	0.23
Sociable	0.79	0.78	0.73
Nervous	0.47	0.61	0.53
Expectant	0.73	0.66	0.74
Hesitant	0.61	0.82	0.46
Trustful	0.54	0.56	0.44
Aggressive	0.40	0.46	0.25
Energetic	0.67	0.62	0.70
Frustrated	0.53	0.70	0.39
Curious	0.66	0.79	0.72
Calming	0.32	0.47	0.53
Indifferent	0.54	0.82	0.54
Stressed	0.52	0.78	0.46

During the following week, the three students who did their final year project on QBA, practiced the method by direct observations of privately owned dogs. 2 weeks after the video scoring, the students applied their QBA skills by scoring during direct observations of dogs in a local shelter. However, the shelter was subsequently closed for intake of new dogs due to an outbreak of acute haemorrhagic diarrhoea of dogs in Norway [14]. For biosecurity reasons, the scoring sessions could therefore not continue. For the direct observations, the inter-observer reliability was found to be high (0.85 for PC1 and 0.76 for PC2), but due to the low sample size (n = 10), these assessments were considered as practice and calibration.

Fifteen months after the first video scoring session, the three students scored the videos a second time (Part II). The descriptors were not discussed before watching the videos, and no further instructions were given. The same 12 video clips were shown, but the order was changed using random number allocation. Because of strict regulation of social distancing due to the COVID-19 pandemic, it was not possible to score the videos under the same conditions as in the first session (i.e., in the same room). The video-scorings were therefore performed during a Microsoft Teams meeting, where the students watched the videos simultaneously on their computers.

Visual analogue scales (VAS) ranging from *Minimum* to *Maximum* were used to score the intensity of each behavioural expression. QBA scores were registered by measuring the distance in millimetres between the *Minimum* point of each VAS, to the point where the scale was ticked by the observer. The data were then transferred to a spreadsheet (Microsoft Office Excel^®^ 2010). Statistical analyses were conducted in Stata SE/16.1 (StataCorp, College Station, Texas). The QBA data were analysed using principal component analysis (PCA) with a correlation matrix (no rotation). PCA reveals the underlying structure of the data and reduces the number of variables to a few main components, each comprising correlated behavioural expressions [12]. To assist in the determination of the number of components to retain, we used a combination of the scree plot criterion and Kaiser’s criterion [15]. The components that explained most of the variance in the data were retained (PC1, PC2), and component scores were calculated. Kendall’s coefficient of concordance (*W*) was used to assess observer reliability. Inter-observer reliability was assessed for the component scores as well as the scores for each individual behavioural descriptor. Intra-observer reliability was calculated for the component scores only, with data from the two different time points (Part Ib and Part II). The reliability coefficients were interpreted according to Martin and Bateson [8].

**Fig. 1:**
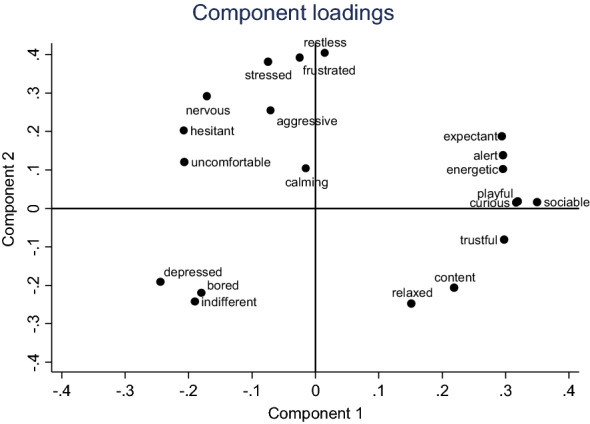
Loading plot depicting how the behavioural terms load along the two main dimensions identified by the principal component analyses of data from Part Ia (12 videos scored by 9 observers)

PCA of the data from the nine participants in Part Ia resulted in a two-component solution, explaining 34.8% and 21.9% of the variance, respectively. PC1 ranged from *depressed, uncomfortable, hesitant,* and *indifferent* to *sociable, curious, playful,* and *trustful*. PC2 ranged from *relaxed, indifferent,* and *content* to *restless, frustrated,* and *stressed* (Fig. 1). *W* for the first (PC1) and second (PC2) component were 0.78 and 0.85, respectively, indicating high agreement (Table 1).

**Fig. 2:**
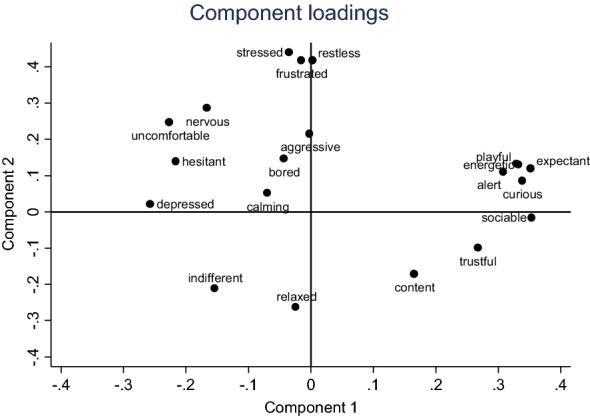
Loading plot depicting how the behavioural terms load along the two main dimensions identified by the principal component analyses of data from Part II (12 videos scored by 3 observers)

PCA of the data from the three observers in Part II resulted in a two-component solution, explaining 30.0 and 20.0% of the variance, respectively. The anchoring points for the principal components were similar to the anchoring points in Part I (Fig. 2), and comparable to the previous study [9]. The first component (PC1) in both parts of the study reflects the dogs’ mood, while the second component (PC2) appears to be related to arousal. *W* for the first component score (PC1) was 0.90, indicating a very high inter-observer agreement. The second component (PC2) had a reliability coefficient of 0.65, indicating moderate agreement (Table 1). The intra-observer agreement was very high for PC1 (*W* > 0.9), and high for PC2 (*W* ≥ 0.86) for all three observers (Table 2).

**Table 2:** Intra-observer agreement for individual observers given as Kendall’s coefficient of concordance (*W*)

Observer	PC1		PC2	
	*W*	p	*W*	p
Student 1	0.92	0.04	0.86	0.06
Student 2	0.93	0.04	0.87	0.06
Student 3	0.93	0.04	0.92	0.04

The high agreement among the observers is in accordance with previous studies [9, 11], however, there were varying levels of reliability of the individual behavioural terms. The nine assessors in Part Ia achieved somewhat lower agreement compared to the three assessors in Part Ib (Table 1). These three students had read six scientific papers on QBA of dogs, sheep, and broiler chickens prior to the scoring session, and this may have improved their understanding of the approach.

The inter-observer reliability of PC1 and PC2 in Part II were high and moderate, respectively, while the intra-observer reliability was high for both PC1 and PC2. There is a risk of observer drift over time, i.e., the observers unconsciously alter their personal understandings of the descriptors. The observers may have acquired different experiences in the time between the scoring sessions, which may have modified their assessments of the animals’ behavioural expressions. Our results suggest that even though the observers were still familiar with the method, and the observer reliability was mainly high, training and calibration sessions are important to avoid observer drift when there is a prolonged period between assessments.

Minero et al. [16] demonstrated that training of observers improves the inter-observer reliability of QBA of donkeys. However, studies addressing the impact of observer training on long-term inter- and intra-observer reliability of QBA assessments appear to be lacking. Bokkers et al. [17] found insufficient observer reliability when experienced observers scored QBA of dairy cattle 9 months after the initial assessments. The authors proposed that one explanation could be that the observers were not trained well enough. The three students participating in Part I and II undertook additional training, practice, and calibration. The combination of video scoring and direct observations associated with training may have been beneficial with regards to the inter- and intra-observer reliability of QBA in this setting.

Our results align with the previous study [9] and suggest that observers can score shelter dogs’ behavioural expressions consistently over time using QBA. Nevertheless, the reduced inter-observer reliability of PC2 in Part II indicates that some degree of retraining may be required to maintain good reliability. The results found in Part II of the study highlight that training, practice and calibration may play a role, and future research may shed more light on this.

## Data Availability

The datasets used and/or analysed during the current study are available from the corresponding author on reasonable request.
